# A Traditional Diet Is Associated with a Reduced Risk of Eczema and Wheeze in Colombian Children

**DOI:** 10.3390/nu7075098

**Published:** 2015-06-25

**Authors:** Alfonso M. Cepeda, Stefano R. Del Giacco, Sara Villalba, Elmy Tapias, Rodolfo Jaller, Ana María Segura, Gloria Reyes, James Potts, Vanessa Garcia-Larsen

**Affiliations:** 1 Fundación Hospital Universitario Metropolitano de Barranquilla, Barranquilla KRA 42F No. 75B-18, Colombia; E-Mails: alerlab@yahoo.es (A.M.C.); saravivar@yahoo.es (S.V.); elmtapias@yahoo.es (E.T.); rodolfojaller@hotmail.com (R.J.); asegurar25@yahoo.es (A.M.S.); gloreyesc@hotmail.com (G.R.); 2 Department of Medical Sciences, University of Cagliari, Cagliari 09042, Italy; E-Mail: delgiac@gmail.com; 3 Respiratory Epidemiology and Public Health Group, National Heart & Lung Institute, Imperial College London, London SW3 6LR, UK; E-Mail: j.potts@imperial.ac.uk; 4 Royal Brompton Hospital and Harefield NHS Foundation Trust, London SW3 6NP, UK

**Keywords:** asthma, eczema, fast food, fruits, pulses, Colombia, diet, ISAAC

## Abstract

Background: Diet might influence the risk of allergic diseases. Evidence from developing countries with high prevalence of childhood asthma is scant. Methods: Information on wheeze, rhinitis, and eczema was collected from 3209 children aged 6–7 years in 2005, who were taking part in the International Study on Asthma and Allergy in Children (ISAAC) in Colombia. Intake frequency of twelve food groups was assessed. Associations between each food group and current wheeze, rhino-conjunctivitis, and eczema were investigated with multiple logistic regressions, adjusting for potential confounders. Simes’ procedure was used to test for multiple comparisons. Results: 14.9% of children reported wheeze in the last 12 months, 16% rhino-conjunctivitis, and 22% eczema. Eczema was negatively associated with consumption of fresh fruits and pulses three or more times per week (adjusted Odds ratio (aOR): 0.64; 95% Confidence Interval (CI): 0.49 to 0.83; *p* value = 0.004; and aOR: 0.62, 95% CI: 0.47 to 0.80; *p* value < 0.001, respectively). Current wheeze was negatively associated with intake of potatoes (aOR: 0.44, 95% CI: 0.31 to 0.62, *p* value = 0.005), whilst this outcome was positively associated with consumption of fast food (aOR: 1.76, 95% CI: 1.32 to 2.35, *p* value = 0.001). These associations remained statistically significant after controlling for multiple comparisons. Conclusions: A traditional diet might have a protective effect against eczema and wheeze in Colombian children, whilst intake of fast foods increases this risk.

## 1. Introduction

The burden of asthma in Latin America represents a major public health challenge, yet research into its possible causes in this region is still scarce [1]. Worldwide cross-sectional evidence from ISAAC (The International Study on Asthma and Allergy in Children) shows that childhood asthma continues to increase in developing countries including those in Latin America, as opposed to the plateau or decline observed in English-speaking nations [2]. Such increasing rates have been associated with poverty, urbanisation and modernisation of habits [3], including the adoption of “Western” lifestyles, changes in dietary habits, obesity, and a more sedentary life.

Oxidative stress and inflammation are central events in the clinical expression of asthma. Diet provides a significant number of components with antioxidant and anti-inflammatory properties which might contribute to lessening the risk of asthma or its severity. Epidemiological evidence shows that children with high adherence to a Mediterranean diet are less likely to suffer from asthma [4]. In Latin America, the role of diet on childhood asthma has been seldom explored, but emerging evidence from Mexican schoolchildren shows that adherence to a healthy diet was associated with a lower risk of asthma and allergic symptoms [5]. Similarly, a case-control study showed that a higher fruit intake may reduce the risk of persistent asthma [6].

Understanding the relationship between diet and asthma in children from developing countries offers a unique opportunity for low-cost and effective public health advice, in nations where over 30% of the population lives in poverty. Using data collected within ISAAC Phase III, this study aimed to investigate the association between outcomes of asthma and allergy and dietary habits in a representative sample of Colombian children.

## 2. Methods

### 2.1. Sample Selection

From January to November 2005, children aged 6–7 years old from 56 primary schools from the cities of Barranquilla and Soledad, in Colombia, were randomly selected to take part in the third phase of the ISAAC study [7]. The schools were selected from the official list of primary schools provided by the Ministry of Education in Colombia. Permission was sought from each school to visit the classroom and deliver the questionnaires to the children. They were asked to take the questionnaire home and to answer it with their mothers, and to return the questionnaire to the school the following day. A member of the research team collected the questionnaires and the data was subsequently tabulated and sent to the ISAAC International Data Centre, Department of Paediatrics, Child and Youth Health, Faculty of Medical and Health Sciences, The University of Auckland, New Zealand where data were quality controlled.

### 2.2. Outcome Assessment

The ISAAC core questionnaire for asthma, rhinitis and eczema was used to ascertain prevalence of each of the following outcomes: For “Ever wheeze”, parents were asked to answer “yes” or “no” to the question “Has your child ever had wheezing or whistling in the chest at any time”? If the answer was “yes”, parents were asked to answer “yes” or “no” to the question “Has your child had wheezing or whistling in the chest in the past 12 months?” (“current wheeze”). Asthma severity was ascertained with the question “How many attacks of wheezing has your child had in the past 12 months?”; “In the past 12 months, how often on average, has your child sleep been disturbed due to wheezing?”; and “In the past 12 months, has wheezing ever been severe enough to limit your child’s speech to only one or two words at a time between breaths?” Rhinitis was considered to be present if the parents answered “yes” to the question “Has your child had a problem with sneezing or a runny or blocked nose when they did not have a cold or flu?” Severe rhinitis was considered present if the parents answered “yes” to the question “In the past 12 months, has this nose problem been accompanied by itchy-watery eyes?” and by answering “a lot” to their nose problems interfering with their daily activities, in the last 12 months. Current eczema was considered present if parents answered “yes” to the question “has your child had an itchy rash at any time during the last 12 months” and answering “yes” to the question “has this itchy rash at any time affected any of the following places: the folds of the elbows, behind the knees, in front of the ankles, under the buttocks, or around the neck, ears or eyes?”. Severe eczema was considered present if the child was reported to have “sleep disturbance one or more times per week due to symptoms of eczema, in the last 12 months”.

### 2.3. Dietary Exposures Assessment

Frequency of intake of major food groups was estimated using the question “In the past 12 months, how often, on average, did your child eat or drink the following foods?: Seafood (including fish), meat (e.g., beef, lamb, chicken, pork), fresh fruit, fresh vegetables (green and root), pulses (peas, beans, lentils), cereals (including bread), pasta, rice, butter, margarine, nuts, potatoes, milk, eggs, fast food/burgers. Parents were asked to choose one of the following frequency options: “Never or occasionally”, “once or twice per week”, and “three or more times a week”.

### 2.4. Statistical Analyses

Descriptive statistics are presented as number and percentages. Prevalence of outcomes is presented as percentages. Associations between outcomes and dietary exposures were investigated with uni- and multivariate regressions. Adjusted regressions included the following potential confounders: maternal educational level, current maternal smoking, maternal smoking during the first year of life, and physical activity of the child. Data were analysed using STATA 12.1 (StataCorp LP 4905 Lakeway Drive College Station, TX, USA).

### 2.5. Ethical Approval

The study protocol was approved by the Ethical Committee of the Metropolitan University of Barranquilla.

## 3. Results

From a total of 3237 children who were given the ISAAC questionnaire, 3209 (99.1%) responded. General characteristics of the sample distribution are presented in Table 1. 47% of the children were males. Over two thirds of the children came from homes where the mother had completed secondary education. Maternal smoking during the child’s first year of life and at the time of the study was rare. The majority of the children were breastfed. Practicing physical exercise at least three times a week was observed in less than half of the children.

**Table 1:** General characteristics of 6–7 year old children participating in ISAAC Phase III Barranquilla.

Variables	Prevalence *N* (%)
**General characteristics**	
Males	1521 (47.3)
Females	1688 (52.7)
Mothers with secondary education (%)	2125 (66.2)
Current maternal smoking	194 (6.0)
Maternal smoking during 1st year of child’s life	161 (5.0)
Child’s physical activity (3 times per week)	1036 (32.3)
3 or more hours per day watching TV	1282 (40.0)
Breastfeeding	2764 (86.1)
**Respiratory and allergic symptoms**	
Ever wheeze	725 (22.6)
Current wheeze (last 12 months)	477 (14.9)
**Severe symptoms of asthma**	
(Answering “yes” to having 4 or more wheeze attacks or being awoken 1 or more nights per week due to wheeze, in the last 12 months)	22 (0.7)
**Rhino-conjunctivitis**	
(Answering “yes” to the question “has your child has a problem with sneezing or a runny or blocked nose when they did not have a cold or flu?” and “In the past 12 months, has this nose problem been accompanied by itchy watery eyes?”)	494 (16.1)
**Severe rhino-conjunctivitis**	
(Having itchy watery eyes and who answered “a lot” to their nose problems interfering with their daily activities, in the last 12 months)	34 (1.1)
**Eczema**	
(Has your child had this itchy rash at any time in the past 12 months? And “has this itchy rash at any time affected any of the following places: the folds of the elbows, behind the knees, in front of the ankles, under the buttocks, or around the neck, ears or eyes?”)	675 (22.3)
**Severe eczema**	
(Having sleep disturbance one or more times per week due to symptoms of eczema, in the last 12 months)	89 (2.9)

**Table 2:** Association between intake of foods rich in proteins or fat and symptoms of asthma and allergy in participating in ISAAC Phase III Barranquilla (Odds Ratios, 95% Confidence intervals, and *p-*values per trend; bold values indicate a statistically significant *p*-value).

Food Group	Current Wheeze		Rhino-Conjunctivitis		Eczema	
	Univariate	Multivariate	Univariate	Multivariate	Univariate	Multivariate
**Meat**						
Never or rarely	Reference	Reference	Reference	Reference	Reference	Reference
Once or twice per week	0.83 (0.49 to 1.39)	0.80 (0.47 to 1.34)	0.69 (0.42 to 1.13)	0.71 (0.43 to 1.16)	1.09 (0.70 to 1.70)	1.10 (0.71 to 1.73)
≥3 times per week	0.82 (0.50 to 1.35)	0.79 (0.48 to 1.30)	0.70 (0.44 to 1.12)	0.71 (0.44 to 1.14)	0.87 (0.57 to 1.34)	0.88 (0.57 to 1.36)
*p-*value	0.59	0.52	0.43	0.41	0.53	0.53
**Seafood**						
Never or rarely	Reference	Reference	Reference	Reference	Reference	Reference
Once or twice per week	0.96 (0.77 to 1.20)	0.96 (0.77 to 1.20)	0.96 (0.77 to 1.19)	0.93 (0.75 to 1.16)	0.87 (0.73 to 1.05)	0.86 (0.72 to 1.04)
≥3 times per week	1.29 (0.96 to 1.73)	1.29 (0.96 to 1.73)	1.17 (0.88 to 1.57)	1.13 (0.84 to 1.51)	0.89 (0.69 to 1.14)	0.87 (0.68 to 1.12)
*p-*value	0.21	0.21	0.47	0.67	0.19	0.14
**Butter**						
Never or rarely	Reference	Reference	Reference	Reference	Reference	Reference
Once or twice per week	0.89 (0.69 to 1.13)	0.88 (0.69 to 1.13)	0.94 (0.73 to 1.20)	0.94 (0.73 to 1.40)	0.91 (0.74 to 1.11)	0.91 (0.74 to 1.11)
≥3 times per week	0.87 (0.67 to 1.13)	0.87 (0.67 to 1.13)	1.08 (0.83 to 1.40)	1.08 (0.83 to 1.40)	0.71 (0.57 to 0.88)	0.71 (0.57 to 0.88)
*p-*value	0.29	0.30	0.53	0.55	**0.002**	**0.002**
**Margarine**						
Never or rarely	Reference	Reference	Reference	Reference	Reference	Reference
Once or twice per week	1.09 (0.85 to 1.39)	1.10 (0.86 to 1.41)	1.10 (0.86 to 1.40)	1.11 (0.87 to 1.41)	1.27 (1.04 to 1.55)	1.26 (1.03 to 1.54)
≥3 times per week	0.93 (0.70 to 1.22)	0.92 (0.70 to 1.22)	0.95 (0.72 to 1.24)	0.91 (0.69 to 1.20)	0.80 (0.63 to 1.01)	0.76 (0.61 to 0.96)
*p-*value	0.72	0.73	0.83	0.68	0.21	0.11
**Milk**						
Never or rarely	Reference	Reference	Reference	Reference	Reference	Reference
Once or twice per week	0.69 (0.43 to 1.13)	0.74 (0.45 to 1.21)	0.71 (0.44 to 1.14)	0.73 (0.45 to 1.19)	0.57 (0.39 to 0.85)	0.57 (0.39 to 0.85)
≥3 times per week	0.66 (0.42 to 1.03)	0.68 (0.43 to 1.06)	0.61 (0.39 to 0.95)	0.64 (0.41 to 0.99)	0.32 (0.22 to 0.47)	0.33 (0.23 to 0.47)
*p-*value	0.13	0.11	**0.03**	**0.04**	**< 0.001**	**< 0.001**
**Eggs**						
Never or rarely	Reference	Reference	Reference	Reference	Reference	Reference
Once or twice per week	1.32 (0.86 to 2.01)	1.31 (0.86 to 2.01)	0.82 (0.57 to 1.19)	0.83 (0.69 to 0.99)	0.74 (0.55 to 1.01)	0.74 (0.54 to 1.01)
≥3 times per week	1.39 (0.90 to 2.13)	1.40 (0.91 to 2.16)	0.95 (0.66 to 1.39)	0.97 (0.67 to 1.42)	0.73 (0.53 to 1.00)	0.73 (0.53 to 1.00)
*p-*value	0.21	0.17	0.51	0.50	0.17	0.15

**Table 3:** Association between fruit and vegetable intake and symptoms of asthma and allergy in participating in ISAAC Phase III Barranquilla (Odds Ratios, 95% Confidence intervals, and *p-*values per trend; bold values indicate a statistically significant *p*-value).

Food Group	Current Wheeze		Rhino-Conjunctivitis		Eczema	
	Univariate	Multivariate	Univariate	Multivariate	Univariate	Multivariate
**Fruits**						
Never or rarely	Reference	Reference	Reference	Reference	Reference	Reference
Once or twice per week	0.96 (0.69 to 1.35)	0.99 (0.71 to 1.39)	0.82 (0.59 to 1.14)	0.86 (0.62 to 1.19)	0.67 (0.51 to 0.88)	0.68 (0.52 to 0.90)
≥3 times per week	0.86 (0.62 to 1.19)	0.87 (0.63 to 1.21)	0.87 (0.64 to 1.19)	0.89 (0.65 to 1.22)	0.63 (0.49 to 0.82)	0.64 (0.49 to 0.83)
*p-*value	0.25	0.26	0.68	0.69	**0.003**	**0.004**
**Vegetables**						
Never or rarely	Reference	Reference	Reference	Reference	Reference	Reference
Once or twice per week	1.32 (0.99 to 1.75)	1.32 (0.99 to 1.75)	0.93 (0.70 to 1.24)	0.92 (0.69 to 1.23)	0.89 (0.71 to 1.12)	0.89 (0.71 to 1.12)
≥3 times per week	1.06 (0.81 to 1.40)	1.07 (0.81 to 1.41)	1.22 (0.94 to 1.58)	1.23 (0.95 to 1.60)	0.89 (0.72 to 1.09)	0.89 (0.72 to 1.10)
*p-*value	0.97	0.99	0.06	0.05	0.32	0.35
**Pulses**						
Never or rarely	Reference	Reference	Reference	Reference	Reference	Reference
Once or twice per week	1.03 (0.74 to 1.43)	1.03 (0.74 to 1.43)	1.03 (0.75 to 1.42)	1.04 (0.75 to 1.44)	0.85 (0.66 to 1.10)	0.86 (0.66 to 1.11)
≥3 times per week	0.88 (0.63 to 1.22)	0.88 (0.63 to 1.23)	0.94 (0.67 to 1.30)	0.95 (0.68 to 1.32)	0.61 (0.47 to 0.80)	0.62 (0.47 to 0.80)
*p-*value	0.23	0.24	0.48	0.53	**< 0.001**	**< 0.001**
**Nuts**						
Never or rarely	Reference	Reference	Reference	Reference	Reference	Reference
Once or twice per week	0.92 (0.71 to 1.19)	0.89 (0.68 to 1.16)	0.93 (0.72 to 1.20)	0.92 (0.71 to 1.19)	0.88 (0.71 to 1.09)	0.86 (0.69 to 1.07)
≥3 times per week	1.04 (0.73 to 1.48)	0.97 (0.68 to 1.39)	0.97 (0.68 to 1.40)	0.91 (0.64 to 1.31)	0.75 (0.55 to 1.02)	0.72 (0.53 to 0.99)
*p-*value	0.89	0.59	0.69	0.49	**0.04**	**0.02**
**Potato**						
Never or rarely	Reference	Reference	Reference	Reference	Reference	Reference
Once or twice per week	0.41 (0.30 to 0.59)	0.41 (0.29 to 0.58)	0.62 (0.43 to 0.89)	0.62 (0.43 to 0.89)	0.52 (0.39 to 0.71)	0.53 (0.39 to 0.72)
≥3 times per week	0.44 (0.31 to 0.62)	0.44 (0.31 to 0.62)	0.63 (0.44 to 0.91)	0.63 (0.43 to 0.91)	0.54 (0.40 to 0.74)	0.54 (0.40 to 0.75)
*p-*value	**0.005**	**0.007**	0.15	0.15	**0.03**	**0.03**

**Table 4:** Association between sources of carbohydrates and symptoms of asthma and allergy in participating in ISAAC Phase III Barranquilla (Odds Ratios, 95% Confidence intervals, and *p-*values per trend; bold values indicate a statistically significant *p*-value).

Food Group	Current Wheeze		Rhino-Conjunctivitis		Eczema	
	Univariate	Multivariate	Univariate	Multivariate	Univariate	Multivariate
**Cereals**						
Never or rarely	Reference	Reference	Reference	Reference	Reference	Reference
Once or twice per week	1.01 (0.67 to 1.53)	1.01 (0.66 to 1.52)	1.08 (0.71 to 1.67)	1.06 (0.69 to 1.63)	1.07 (0.76 to 1.52)	1.05 (0.75 to 1.49)
≥3 times per week	0.87 (0.59 to 1.29)	0.87 (0.58 to 1.28)	1.12 (0.75 to 1.68)	1.09 (0.72 to 1.64)	0.91 (0.66 to 1.27)	0.90 (0.65 to 1.25)
*p-*value	0.19	0.20	0.58	0.64	0.16	0.14
**Pasta**						
Never or rarely	Reference	Reference	Reference	Reference	Reference	Reference
Once or twice per week	0.90 (0.67 to 1.21)	0.90 (0.68 to 1.21)	1.03 (0.76 to 1.39)	1.01 (0.74 to 1.36)	1.02 (0.81 to 1.31)	1.00 (0.79 to 1.28)
≥3 times per week	0.95 (0.70 to 1.31)	0.97 (0.70 to 1.33)	1.23 (0.89 to 1.70)	1.22 (0.88 to 1.69)	0.87 (0.67 to 1.14)	0.85 (0.65 to 1.11)
*p-*value	0.92	0.98	0.11	0.12	0.19	0.14
**Rice**						
Never or rarely	Reference	Reference	Reference	Reference	Reference	Reference
Once or twice per week	0.50 (0.30 to 0.83)	0.51 (0.30 to 0.85)	0.60 (0.36 to 1.00)	0.63 (0.37 to 1.10)	0.47 (0.31 to 0.72)	0.48 (0.31 to 0.75)
≥3 times per week	0.59 (0.37 to 0.92)	0.59 (0.37 to 0.93)	0.60 (0.38 to 0.95)	0.63 (0.39 to 1.00)	0.44 (0.30 to 0.65)	0.45 (0.31 to 0.67)
*p-*value	0.37	0.35	0.12	0.17	**0.001**	**0.002**
**Fast food**						
Never or rarely	Reference	Reference	Reference	Reference	Reference	Reference
Once or twice per week	1.12 (0.89 to 1.41)	1.11 (0.88 to 1.40)	1.07 (0.86 to 1.33)	1.09 (0.87 to 1.35)	1.03(0.86 to 1.23)	1.02(0.85 to 1.23)
≥3 times per week	1.76 (1.32 to 2.35)	1.74 (1.30 to 2.34)	1.11 (0.81 to 1.51)	1.11 (0.81 to 1.51)	1.11 (0.86 to 1.43)	1.09 (0.84 to 1.40)
*p-*value	**0.001**	**0.001**	0.48	0.44	0.46	0.55

The prevalence of current wheeze (in the last 12 months) reached 15% in this population, whilst ever wheeze was slightly higher. Rhino-conjunctivitis affected 16% of children, whilst eczema was reported in 22% of the population. Symptoms of asthma severity were less common, with only 0.7% reporting having severe symptoms of asthma, 1.1% severe rhinoconjunctivitis, and 2.9% severe asthma.

The association between dietary intake of foods rich in animal proteins and respiratory and allergic outcomes is illustrated in Table 2. After adjustment for potential confounders, dietary intake of butter, but not margarine, was negatively associated with eczema (OR: intake 3+ times per week *vs*. never: 0.71; 95% CI: 0.57 to 0.88). Similarly, a regular intake of milk was negatively associated with eczema (OR: 0.33; 95% CI: 0.23 to 0.47). A weaker negative association was also observed between milk and rhino-conjunctivitis. Regular consumption of fruits and pulses was negatively associated with eczema, whilst a weaker negative association was observed between nuts and potatoes with this outcome (Table 3). Regular intake of potatoes was also negatively associated with current wheeze (*p* = 0.007). Intake of rice was also negatively associated with eczema, whilst intake of fast food three or more times per week was statistically significantly associated with current wheeze (OR: 1.74; 95% CI: 1.30 to 2.34) (Table 4).

## 4. Discussion

As part of the ISAAC worldwide study, we found in this cross-sectional analysis of a representative sample of children from Colombia a statistically significant negative association between eczema and milk, butter, rice, and pulses, which are staple foods in many South American countries. Eczema was also negatively associated with a higher intake of fruit, whilst current wheeze was positively associated with intake of fast food. These associations remained statistically significant after controlling for multiple testing.

Diet contains a wealth of components with antioxidant or anti-inflammatory properties, which have been proposed to counteract the cascade of events that lead to the asthmatic response [8]. In this analysis, children who consumed milk three or more times per week had a significantly lower risk of having eczema. Milk is a rich source of antioxidant compounds including selenium and casein, which contribute to most of its antioxidant capacity [9]. Several reasons might explain our findings. The ISAAC questionnaire was not designed to capture intake of raw or pasteurised milk, but it is possible that a significant proportion of the participant children consumed raw milk. Colombia has a strong agricultural economy and intake of unpasteurised milk is very common in the country [10], and it is cheaper than pasteurised milk. The children in our study were mostly from urban areas. However, the possible protective effect that unpasteurized milk would appear have has been shown to be independent of whether children are raised in a farm environment [11].

A lower risk of eczema was also associated with a higher intake of butter, but not margarine. In contrast to margarine, butter does not normally contain pro-inflammatory omega 6 *trans*-fatty acids, which have been proposed to influence the development of allergic diseases in children [12]. In South America, consumption of margarine has been recommended as a preferred option over butter to reduce the risk of cardiovascular disease [13]. Colombian margarines, in particular, have been described to have a lower *trans*-fatty acid content and higher levels of saturated fatty acids than the levels observed in margarines from other Latin American countries [14]. It might be possible that the lower content of *trans*-fatty acids makes margarine unlikely to have a detrimental effect on allergic symptoms in the children studied in Barranquilla. The variations in margarine fatty acid consumption might also explain the higher risk reported in the ISAAC children from the Asia Pacific region [15] and Hungary [16]. Other observational studies in Europe have found no association between butter intake in the first years of life or at age 8 and eczema in Dutch children [17].

Pulses are excellent sources of niacin, zinc, and folic acid. In South America, these grains are central to the dietary habits of the population. Pulses are also good sources of isoflavonoid, which are widely found in foods of the *Leguminosae* family [18]. Children with atopic dermatitis and eczema-like symptoms have been shown to have significantly lower levels of folic acid, zinc and niacin compared with healthy matches [19]. Interventions with zinc given to children with atopic dermatitis have been shown to reduce the severity of pruritus after 8 weeks of supplementation [20].

In our study we also found a strong negative association between eczema and fruit intake. Fruits are rich in many compounds with antioxidant activity and might impact the inflammatory pathway that leads to the expression of eczema. The epidemiological evidence in this area is mixed. Exposure to fruit intake during pregnancy has been found to have a protective effect against risk of eczema in the offspring [21] although other cohort studies have not confirmed these associations [22]. Our results have been confirmed in other cross-sectional surveys. In ISAAC children from Spain, a Mediterranean diet (containing fruit as part of a diet rich in vegetables, olive oil and fish) was associated with a lower risk of atopic eczema [23]. The authors built a score according to the frequency of intake (daily, sometimes, never) of the foods listed in the ISAAC questionnaire. This Mediterranean score was based on the usual diet of the Spanish population. Whether such a Mediterranean pattern applies to Colombia or to South America is unclear, and as a first step to investigate the association between foods and allergic outcomes, we used the original grouping of food variables used in the ISAAC questionnaire.

Intake of fast food was positively associated with the risk of wheeze in in our study. Although the content and definition of fast food might vary across countries, the term usually includes consumption of burgers and foods rich in simple sugars (e.g., sweets, fizzy drinks). The nutritional content of these foods tend to be poor, with a high caloric density derived mostly from fats and sugars. The role of diet in promoting a more pro- or anti-inflammatory environment can affect the microbiota in the gut, which in turns can have a detrimental effect on general immunity and the airways [24]. One of the mechanisms through which processed foods favor a more pro-inflammatory environment is by altering the microbiota balance [25]. The abundance of *trans*- and saturated fatty acids as well as single sugars can facilitate the metabolism of arachidonic acid, and the consequent synthesis of pro-inflammatory cytokines. As Colombia is a developing country with a fast growing economy, it is possible that the early exposure to more “Western” dietary habits is affecting health in young children.

Our findings concerning a positive association between fast food and wheeze are in line with those reported in the worldwide sample collected during ISAAC III. Ellwood and colleagues showed a positive association between severe asthma and intake of fast food in children aged 6–7 years old and with wheeze in adolescents (12–13 years old) [26]. In our study, the prevalence of severe asthma was extremely low (<1%) and therefore we did not examine the association with dietary exposures. Other cross-sectional surveys in Europe have also reported an association between having dietary pattern characterized by high intake of processed foods, with current and Dr.-diagnosed asthma in children aged 8 and 11 years old [27]. Although there are grounds to justify a mechanistic effect of processed or fast foods on asthma, the current observational evidence preclude us from establishing whether fast food is in the causal pathway of asthma. The fact that dietary habits are usually a reflection of other lifestyle factors raises the issue of possible confounding by other relevant exposures that have not been measured [28].

We used a pre-established three-level categorization of frequency of consumption for food groups, as defined in the main ISAAC questionnaire. This has the advantage of giving an indication of those foods that are more commonly consumed, and hence are part of the usual diet, and those foods that are less often consumed. However, like any instrument to ascertain dietary intake, the ISAAC questionnaire is not free from error and could have some recall bias as it relies on the self-reporting information provided by parents (if children are aged 6–7 years old) or in the children themselves when they are older (10–12 years old).

Our findings are cross-sectional and intake of foods was surveyed over the last 12 months, so we are unable to infer causality. The fact that traditional foods of the Colombian diet such as pulses, rice and grains were negatively associated with allergic outcomes might be suggestive of a protective effect of a typical Colombian diet. However, to elucidate whether diet can prevent allergic diseases, these findings would need to be replicated in longitudinal studies. Whether timing of dietary exposure to fruit intake is important in the prevention of eczema is still a controversial issue, as epidemiological evidence is mixed. Some birth cohort studies found that a higher maternal intake of fruit during pregnancy has been related to a lower risk of eczema in infants [21], and that a low intake of fruit in pregnant mothers was positively associated with risk of eczema in children up to the age of 5 years [29]. Other cohorts, however, have not found an effect in maternal exposure and risk at age 2 [30] or 5 years old [31].

Most of the available findings on associations between outcomes of allergic diseases and dietary exposures come from Europe and North America. The ISAAC study has provided the opportunity to assess, in a standardised fashion, the prevalence of childhood asthma and allergic symptoms across continents. Our study shows that allergic diseases are highly prevalent in children from Colombia. The burden of respiratory and allergic diseases in South American children appears to be as high as that observed in more developed continents. In spite of this, there are still very few epidemiological studies in the region investigating the association between asthma, allergy, and lifestyle-related factors such as diet. The available findings so far suggest that dietary sources of flavonoids (e.g., catechins) and vegetables might be beneficial against asthma in young adults [32] and adolescents [33].

## 5. Conclusions

Our study of a representative sample of 6–7 year old children from Barranquilla, Colombia, suggests that children who eat foods that are part of their staple diet have a reduced risk of eczema. Conversely, asthma is more prevalent in those with a more frequent intake of fast food. Our findings offer a foundation for longitudinal and intervention studies to confirm these results, and for public health recommendations to reduce the current burden of these diseases.
